# Impact of Graft Steatosis on Postoperative Complications after Liver Transplantation

**DOI:** 10.1055/s-0038-1675236

**Published:** 2018-10-18

**Authors:** Emad Ali Ahmed, Ashraf Mohammad El-Badry, Federico Mocchegiani, Roberto Montalti, Asem Elsani Ali Hassan, Alaa Ahmed Redwan, Marco Vivarelli

**Affiliations:** 1 Division of Hepatobiliary and Abdominal Transplantation Surgery, Department of Experimental and Clinical Medicine, Polytechnic University of Marche, Ancona, Italy; 2 Division of Hepatobiliary and Pancreatic Surgery Unit, Department of General Surgery, University of Sohag, Sohag, Egypt

**Keywords:** graft steatosis, fatty liver, liver transplantation, macrosteatosis, microsteatosis, reperfusion injury

## Abstract

**Background** Steatotic grafts are more susceptible to ischemia-reperfusion injury than are normal grafts. Therefore, using steatotic grafts for liver transplantation (LT) is associated with high primary dysfunction and decreased survival rates. The aim of this study is to evaluate the impact of graft steatosis on post LT outcomes.

**Methods** A retrospective cohort analysis of 271 LT recipients from 2005 to 2016 was performed and patients were classified based on two types of steatosis, macrosteatosis (MaS), and microsteatosis (MiS). Each category was subdivided into three groups according to the degree of steatosis: no (< 5%), mild (≥5 to < 30%), and moderate (≥30 to ≤60%). The primary hospital stays and 6-month postoperative complications were analyzed by the Clavien–Dindo classification system. Additionally, patient and graft survivals were studied.

**Results** Significant differences were observed in grade III MaS ( *p* -value = 0.019) and grade V MiS ( *p* -value = 0.020). A high trend of early graft dysfunction was found in the moderate MaS and MiS groups; however, they were not statistically significant ( *p* -value = 0.199 and 0.282, respectively). Interestingly, the acute cellular rejection (ACR) rate was found to be inversely proportional to the degree of steatosis in both categories but it did not reach a significant level ( *p* -value = 0.161 and 0.111, respectively).

**Conclusion** Excellent post LT long-term outcomes using grafts with mild and moderate steatosis were determined. Further studies are needed to evaluate the newly proposed relationship between ACR and steatosis.

The number of available grafts for liver transplantation (LT) has failed to keep pace with the needs of the recipient pool of patients. Thus, extending the donor pool remains a critical concern. There are several forms of extended criteria donors who can provide more grafts but the grafts are marginal. Steatosis is considered one of the most widely represented marginal grafts because of the worldwide increasing prevalence of nonalcoholic fatty liver diseases. 1

Liver steatosis is subdivided qualitatively into macrosteatosis (MaS) and microsteatosis (MiS). MaS is characterized by a single fat vacuole in hepatocytes, displacing the nucleus to the edge of the cell. In MiS, the cytoplasm of the hepatocytes contains tiny lipid vesicles without nuclear dislocation. Steatosis is further classified quantitatively into mild, moderate, or severe if (≥5 to < 30%), (≥30 to ≤60%), or (> 60%) of hepatocytes, respectively, display fatty infiltrations. 2

Despite their benign nature, fatty livers may be associated with serious injury including inflammation and hepatocyte necroapoptosis. 3 In the past, grafts with ≥30% fatty infiltration were not acceptable for transplantation. Furthermore, the use of livers with less fatty infiltration (< 30%) has been reported to result in bad outcomes after LT. 4 Recently, some authors reported excellent results after transplantation using severely steatotic livers but with a lower model for end-stage liver disease (MELD) score and shorter ischemia times. 5 6 7 8

Great diversity exists in regard to the impact of the type of steatosis on post LT outcomes. Some authors regard MaS to be more clinically relevant than MiS 9 10 while others consider MiS to be an independent risk factor that can affect the early function of the graft. 11 Cieślak et al observed that a 12% increase in MiS was equivalent to a 50% increased risk for postoperative graft dysfunction. 12

Therefore, our aim was to evaluate the impact of graft steatosis on post LT outcomes.

## Methods

### Study Design

This study is a retrospective cohort study that was a collaboration between Sohag University, Egypt and Polytechnic University of Marche, Italy. Ethical committee approval for the study was obtained with the No. 0113150/02–14. All patients provided informed written consent prior to the transplant procedures and transplant waiting list inclusion. The study protocol was registered at ClinicalTrials.gov as NCT02659553. The study was performed utilizing the “Strengthening the Reporting of Cohort Studies in Surgery (STROCSS)” statement. 13

### Setting and Participants

The study was conducted at the Division of Hepatobiliary and Abdominal Transplantation Surgery, Department of Experimental and Clinical Medicine, Polytechnic University of Marche, Ancona, Italy. The study included the transplanted cases from August 2005 to April 2016 that matched the eligibility criteria.

#### Eligibility Criteria

All adult patients who underwent primary LT with a preoperative histopathological report of the donor graft determining the type and degree of fatty infiltration were included in this study. Retransplanted patients and patients with no preoperative determination of the type and/or degree of graft steatosis were excluded from the study.

#### Data Collection

The patients' records of 271 recipients that matched our eligibility criteria were analyzed for any postoperative complications during the primary hospital stay (from the date of LT to the date of discharge). Overall complications during the first 6 postoperative months and long-term patient and graft survivals were also studied.

The severity of the liver disease of the recipients was assessed by MELD score. ABO blood group compatible grafts from deceased heart-beating donors with accepted liver function tests were used for all participants. Organ procurement was performed according to the standard techniques with the back-table preparation of the graft. A graft biopsy was obtained using a true-cut needle at the time of procurement. Grafts were preserved using Celsior ( *n* = 262), University of Wisconsin ( *n* = 8), or Histidine-Tryptophan-Ketoglutarate ( *n* = 1) solutions and placed on ice.

Recipient total hepatectomy followed by LT using the conventional ( *n* = 19), piggyback ( *n* = 247), or piggyback variant ( *n* = 5) technique was performed. Then, end-to-end porto-portal vein (porto-PV) anastomosis ( *n* = 266), the cavoportal hemitransposition technique ( *n* = 3), or bypass porto-mesenteric anastomosis ( *n* = 2) was performed. The hepatic arterial circulation was maintained through end-to-end hepatic artery anastomosis ( *n* = 247), celiac–hepatic ( *n* = 6), celiac–celiac ( *n* = 2), celiac–aortic ( *n* = 6), or hepatic–aortic anastomosis ( *n* = 10). Biliary reconstruction was performed by end-to-end choledochocholedochostomy ( *n* = 257) or hepaticojejunostomy ( *n* = 14).

In our institution, 500 mg of steroids plus 20 mg of basiliximab were administered at the time of perfusion. Then, postoperative immunosuppressive schemes were tailored for each patient but they were predominantly based on steroids, tacrolimus, and/or basiliximab.

During the hospital stay, postoperative graft follow-up was achieved by daily measurements of transaminases, albumin, bilirubin, and the international normalized ratio (INR). A duplex examination was performed routinely every day for the first postoperative week, then every other day until hospital discharge, then monthly for the first year. A postoperative graft biopsy was not routinely done in our center except when there was clinical or biochemical evidence of graft dysfunction.

### Study Variables and Measurements

Steatosis was considered the factor of interest in this study. Qualitatively patients were divided into two categories: MaS or MiS. Each category was subdivided quantitatively into three groups: control group with no steatosis (< 5%), compared with mild (≥5 to < 30%), and moderate (≥30 to ≤60%) steatosis.

These groups were analyzed for any major postoperative negative outcomes during the hospital stay based on the Clavien–Dindo classification with definitions specified for LT. 14 15 Other outcomes of interest included primary nonfunction (PNF), early graft dysfunction (EGD), acute cellular rejection (ACR), biliary complications, and patient and graft survivals.

PNF was defined as graft failure soon after reperfusion without an obvious cause leading to either retransplantation or death in the first postoperative week. It was diagnosed by (1) an increased aspartate transaminase (AST) level ≥3,000 U/L and (2) INR ≥2.5 and/or acidosis (arterial pH ≤7.30 or venous pH ≤7.25 and/or lactate ≥4 mmol/L). 16

EGD was defined as impaired initial graft function with a high peak serum transaminase and persistently high bilirubin levels. It was diagnosed by measurement of one or more of the following values: bilirubin ≥10 mg/dL on day 7, INR ≥1.6 on day 7, and AST > 2,000 IU/L within the first 7 days. 17

ACR was broadly defined as graft inflammation elicited by genetic disparity between the donor and recipient. Graft biopsy, obtained using a true-cut needle, remains the gold standard for ACR diagnosis. 18

Bile leakage is defined as a fluid with an increased bilirubin concentration (three times greater than the serum bilirubin measured at the same time) in the abdominal drain or in the intra-abdominal fluid on or after postoperative day (POD) 3 or as the need for radiologic intervention or relaparotomy. 19 Biliary stricture is an abnormal narrowing of the bile duct associated with rising cholestasis indexes that required invasive management, such as endoscopic retrograde cholangiopancreatography, percutaneous transhepatic drainage, or resurgery. 20

Acute postoperative vascular complications including portal vein thrombosis (PVT), hepatic artery thrombosis (HAT), and haemoperitoneum were studied. Postoperative ascites, renal insufficiency, hepatitis C virus (HCV) recurrence, and intensive care unit (ICU) and hospital stays were also evaluated.

Patient survival was calculated from the date of transplantation to the date of patient death. Graft survival was calculated from the date of transplantation to the date of irreversible graft failure, retransplantation, or the date of death.

### Bias Assessment

All records were independently reviewed by the endpoint assessment committee which included certified surgeons and gastroenterologists. All members of the end point assessment committee were blinded to the study participants' baseline risk factor information.

### Statistical Analysis

Continuous variables were presented as the means and standard deviation while categorical variables were expressed as percentages. Quantitative variables were compared using a one-way ANOVA (analysis of variance) test. For qualitative variables, Pearson's Chi-square tests were used after assumptions had been verified. A 95% confidence interval (CI) was reported for both measures. Graft and patient survival rates were described and compared using a Kaplan–Meier test. A *p* -value < 0.05 was considered statistically significant. All statistical tests were performed using IBM SPSS Statistics for Windows, Armonk, NY: IBM Corp, Version 20.

## Results

### Participants and Descriptive Data

From August 2005 to April 2016, 271 cases matched our inclusion and exclusion criteria and were included in this study. The primary indications for LT in the selected patients are shown in Table 1 and included end-stage liver disease due to hepatitis ( *n* = 178), alcoholism ( *n* = 45), or other ( *n* = 46), and/or HCC (hepatocellular carcinoma; *n* = 103), cholangiocarcinoma ( *n* = 1), or complicated adenoma ( *n* = 1). To the best of our knowledge, all cases of HCC were associated with a different liver pathology. Regarding the type and degree of graft steatosis, 125 patients received grafts with MaS of which 109 were mild and 16 were moderate. Additionally, 96 patients received grafts with MiS of which 77 were mild and 19 were moderate. The clinicopathological features of the donors and recipients are listed in Table 1 .

**Table 1:** Demographic and clinicopathological criteria of all participants

Type of steatosis	Macrosteatosis ( *n* = 271)				Microsteatosis ( *n* = 271)			
Degree of steatosis	No *n* = 146	Mild *n* = 109	Moderate *n* = 16	*p* -Value	No *n* = 175	Mild *n* = 77	Moderate *n* = 19	*p* -Value
Donor criteria ( *n* = 271)								
Age (y)	60.9 ± 17.74	64.3 ± 14.26	57.2 ± 17.37	0.126	61.36 ± 17.00	65.35 ± 13.65	55.00 ± 19.62	0.031
Sex (m/f)	75/71	62/47	10/6	0.540	99/76	38/39	10/9	0.564
D-MELD score	1,100 ± 643	1,127.6 ± 600	740.9 ± 396.9	0.062	1,073.7 ± 625.7	1,182.8 ± 615	863 ± 518.7	0.110
DRI	1.86 ± 0.59	1.95 ± 0.75	1.68 ± 0.38	0.261	1.89 ± 0.64	1.92 ± 0.70	1.60 ± 0.40	0.162
BMI (kg/m ^2^ )	25.38 ± 3.01	27.16 ± 4.25	27.87 ± 4.21	0.000	26.24 ± 3.96	26.28 ± 3.36	26.15 ± 3.28	0.991
ALT (U/L)	47.28 ± 64.06	39.52 ± 42.75	50.4 ± 50.07	0.513	44.61 ± 56.75	40.03 ± 46.39	59.89 ± 77.56	0.401
AST (U/L)	49.8 ± 64.75	47.36 ± 52.9	68.0 ± 93.38	0.490	48.92 ± 62.24	47.45 ± 55.8	68.56 ± 84.54	0.416
PT-INR	3.73 ± 16.9	4.05 ± 17.05	1.21 ± 0.26	0.845	4.89 ± 20.12	1.44 ± 1.26	1.29 ± 0.15	0.327
Na	150.05 ± 9.71	150.2 ± 9.08	148.9 ± 10.9	0.880	149.73 ± 9.48	150.54 ± 9.65	151.12 ± 9.45	0.743
ICU stay (d)	4.67 ± 4.19	4.67 ± 4.35	4.4 ± 3.38	0.972	4.69 ± 4.22	4.73 ± 4.35	4.05 ± 3.55	0.810
Cause of death								
Cerebral hemorrhage	91 (62.3)	65 (59.6)	7 (43.8)	0.868	106 (60.6)	48 (62.3)	9 (47.4)	0.908
Cranial trauma	29 (19.9)	23 (21.1)	7 (43.8)	0.868	36 (20.6)	15 (19.5)	8 (42)	0.908
Brain anoxia	13 (8.9)	10 (9.2)	1 (6.2)	0.868	16 (9)	6 (7.8)	2 (10.5)	0.908
Ischemic stroke	9 (6.2)	9 (8.3)	1 (6.2)	0.868	11 (6.3)	8 (10.4)	0 (0)	0.908
Others	4 (2.7)	2 (1.8)	0 (0)	0.868	6 (3.4)	0 (0)	0 (0)	0.908
Recipient criteria ( *n* = 271)								
Age (y)	53.84 ± 8.35	52.53 ± 9.39	56.75 ± 6.68	0.152	53.62 ± 8.62	53.35 ± 8.99	52.74 ± 9.12	0.905
Sex (m/f)	111/35	83/26	11/5	0.803	131/44	58/19	16/3	0.664
MELD score	17.84 ± 8.25	16.09 ± 7.49	19.50 ± 7.22	0.110	17.34 ± 7.99	17.26 ± 7.56	16.16 ± 9.12	0.828
BMI (kg/m ^2^ )	24.71 ± 3.99	25.1 ± 4.23	25.37 ± 3.54	0.678	25.30 ± 4.25	24.03 ± 3.47	24.84 ± 3.99	0.072
Indications of LT								
HCV	69 (47.3)	54 (49.5)	7 (43.4)	0.830	88 (50.3)	37 (48)	5 (26.3)	0.445
Plus, HCC	55 (37.7)	40 (36.7)	8 (50)	0.830	71 (40.6)	27 (35)	5 (26.3)	0.445
Alcoholic	25 (17)	15 (13.8)	5 (31.3)	0.830	28 (16)	12 (15.6)	5 (26.3)	0.445
Cryptogenic	13 (9)	9 (8.3)	1 (6.3)	0.830	16 (9)	6 (7.8)	1 (5.3)	0.445
HBV	12 (8)	10 (9.2)	2 (12.5)	0.830	11 (6.3)	10 (13)	3 (15.8)	0.445
HBV–HDV	8 (5.5)	9 (8.3)	0 (0)	0.830	12 (6.9)	4 (5.2)	1 (5.3)	0.445
Cholestasis	10 (7)	3 (2.8)	1 (6.3)	0.830	9 (5)	3 (3.9)	2 (10.5)	0.445
Others	9 (6)	9 (8)	0 (0)	0.830	11 (6.3)	5 (6.5)	2 (10.5)	0.445
ALT (U/L)	69.6 ± 94.6	216.9 ± 1047	43.88 ± 31.44	0.202	82.41 ± 175.4	243 ± 1,228	73.37 ± 150	0.212
AST (U/L)	100.9 ± 110	297.5 ± 1410	63.62 ± 48.3	0.205	107.8 ± 159.6	357.7 ± 1663	86.47 ± 97	0.122
PT-INR	5.73 ± 24.16	5.0 ± 24.19	1.81 ± 0.75	0.813	4.81 ± 20.42	6.83 ± 31.42	2.21 ± 2.7	0.695

Abbreviations: ALT, alanine transaminase; AST, aspartate transaminase; BMI, body mass index; D-MELD, donor-model end-stage liver disease; DRI, donor risk index; HBV, hepatitis B virus; HCC, hepatocellular carcinoma; HCV, hepatitis C virus, HDV, hepatitis D virus; ICU, intensive care unit; LT, liver transplantation; PT/INR, prothrombin time/international normalized ratio.

In the MaS category, the only significant difference was in the donor body mass index (BMI) which was lower in the control group than in the mild and moderate groups (25.38 ± 3.01 vs. 27.16 ± 4.25 vs. 27.87 ± 4.21, *p* = 0.000, respectively). Moreover, the D-MELD (donor-MELD) score was much lower in the moderate group than in the control and mild groups but the scores were not significantly different (740.94 ± 396.98 vs. 1100.07 ± 643.3 vs. 1127.6 ± 600.17, *p* = 0.062, respectively).

In the MiS category, the only significant difference was the donor age which was markedly lower in the moderate group than in the control and mild groups (55.00 ± 19.62 vs. 61.36 ± 17.00 vs. 65.35 ± 13.65, *p* = 0.031, respectively).

The operative parameters and ischemia times are listed in Table 2 . No differences were noted among any groups within MaS and MiS in regard to the cold ischemia time (CIT), total ischemia time (TIT), or surgical time.

**Table 2:** Operative parameters of all patients

Type of steatosis	Macrosteatosis ( *n* = 271)				Microsteatosis ( *n* = 271)			
Degree of steatosis	No *n* = 146	Mild *n* = 109	Moderate *n* = 16	*p* -Value	No *n* = 175	Mild *n* = 77	Moderate *n* = 19	*p* -Value
CIT (min.)	433.13 ± 113.46	414.62 ± 121.98	440.56 ± 150.08	0.419	423.16 ± 111.98	428.10 ± 130.68	445.84 ± 138.39	0.724
WIT (min.)	32.51 ± 10.63	43.12 ± 66.63	44.63 ± 20.38	0.123	37.09 ± 45.89	39.13 ± 42.68	34.21 ± 11.09	0.891
TIT (min.)	465.64 ± 114.56	457.74 ± 113.23	485.19 ± 156.5	0.650	460.25 ± 110.26	467.23 ± 124.52	480.05 ± 142.46	0.743
Operative time (min.)	421.68 ± 79.43	416.87 ± 90.88	461.56 ± 108.8	0.154	426.03 ± 90.82	411.16 ± 77.87	430.79 ± 75.62	0.410

Abbreviations: CIT, cold ischemia time; TIT, total ischemia time; WIT, warm ischemia time.

### Outcome Data and Main Results

#### Impact of Graft MaS on Post LT Outcomes

According to the Clavien–Dindo grading of in-hospital complications ( Table 3 ), a significant difference in the grade III complication rate ( *p* = 0.019) was detected among the groups with a higher rate in the moderate group (31%) than in the control (nonsteatotic; 23%) and mild (11%) groups. This difference was also observed between the moderate ( *p* = 0.027) and control ( *p* = 0.012) groups when compared with the mild group separately. No significant difference was noted when comparing the moderate and control groups ( *p* = 0.479). An increase in the grade IV complication rate was also observed in the moderate group (12.5%) compared with the control and mild groups (3.4 and 1.8%, respectively) but the difference did not reach statistical significance ( *p* = 0.084). No significant difference was detected among the groups in regard to the grade V complication rate ( *p* = 0.215).

**Table 3:** Postoperative outcomes after liver transplantation

Type of steatosis	Macrosteatosis ( *n* = 271)				Microsteatosis ( *n* = 271)			
Degree of steatosis	No *n* = 146	Mild *n* = 109	Moderate *n* = 16	*p* -Value	No *n* = 175	Mild *n* = 77	Moderate *n* = 19	*p* -Value
Overall complication								
PNF	4 (2.7)	5 (4.6)	1 (6.2)	0.634	5 (2.9)	5 (6.5)	0 (0.0)	0.250
EGD	49 (33.6)	39 (35.8)	9 (56.2)	0.199	60 (34.3)	27 (35.1)	10 (52.6)	0.282
PVT	2 (1.4)	1 (0.9)	0 (0.0)	0.858	3 (1.7)	0 (0.0)	0 (0.0)	0.435
Acute HAT	4 (2.7)	4 (3.7)	2 (12.5)	0.145	7 (4.0)	3 (3.9)	0 (0.0)	0.676
Ascites	27 (18.5)	14 (12.8)	2 (12.5)	0.441	26 (14.9)	15 (19.5)	2 (10.5)	0.524
Bile leakage	17 (11.6)	14 (12.8)	0 (0.0)	0.319	13 (7.4)	12 (15.6)	2 (10.5)	0.137
Biliary stricture	24 (16.4)	17 (15.6)	3 (18.8)	0.946	27 (15.4)	12 (15.6)	3 (15.8)	0.999
Haemoperitoneum	9 (6.2)	7 (6.4)	1 (6.2)	0.996	9 (5.1)	6 (7.8)	2 (10.5)	0.531
Renal failure	17 (11.6)	8 (7.3)	1 (6.2)	0.460	15 (8.6)	9 (11.7)	2 (10.5)	0.734
ACR	58 (39.7)	35 (32)	3 (18.8)	0.161	68 (39)	25 (32.5)	3 (15.8)	0.111
HCV recurrence	14 (9.6)	9 (8.3)	0 (0.0)	0.423	17 (9.7)	7 (9.1)	0 (0.0)	0.366
Retransplantation	6 (4)	6 (5.5)	0 (0.0)	0.584	8 (4.6)	3 (4)	1 (5.3)	0.955
Hospital stay complications as graded by Clavien–Dindo								
Grade III	34 (23.3)	12 (11)	5 (31)	0.019	34 (19.4)	13 (17)	5 (26.3)	0.640
Grade IV	5 (3.4)	2 (1.8)	2 (12.5)	0.084	6 (3.4)	0 (0)	0 (0)	0.186
Grade V	12 (8.2)	16 (14.7)	1 (6.2)	0.215	14 (8.0)	15 (19.5)	1 (5.3)	0.020
ICU stay (d)	7.81 ± 32.46	5.25 ± 7.45	9.2 ± 9.6	0.696	7.71 ± 29.96	5.62 ± 6.47	4.24 ± 3.47	0.765
Hospital stay (d)	21.43 ± 12.64	20.03 ± 13.44	21.6 ± 10.76	0.719	21.33 ± 13.56	20.15 ± 11.75	19.94 ± 9.11	0.788

Abbreviations: ACR, acute cellular rejection; EGD, early graft dysfunction; HAT, hepatic artery thrombosis; HCV, hepatitis C virus; ICU, intensive care unit; PNF, primary nonfunction; PVT, portal vein thrombosis.

Regarding the overall complications encountered after LT ( Table 3 ), one patient in the moderate group (6.2%), five in the mild group (4.6%) and four in the control group (2.7%) had experienced PNF, with no statistical increase in PNF ( *p* = 0.634). In addition, there was a higher rate of EGD and acute HAT when comparing the moderate group (56.2 and 12.5%, respectively) with the mild (35.8 and 3.7%, respectively) and control (33.6 and 2.7%, respectively) groups. Although there was a large difference in the rates of EGD and HAT, no statistical significance was observed ( *p* = 0.199 and 0.145, respectively). Conversely, the ACR rate was higher in the control and mild groups compared with the moderate group (39.7, 32, and 18.8%, respectively) but it did not reach a significant level ( *p* = 0.161).

Within the first postoperative 6 months, no cases of postoperative bile leakage or HCV recurrence were documented in the moderate group compared with the control (11.6 and 9.6%, respectively) and mild (12.8 and 8.3%, respectively) groups, and there was no significant difference among the groups ( *p* = 0.319 and 0.423, respectively). Nearly equal results were obtained among all MaS groups in regard to the rates of postoperative ascites, biliary stricture, haemoperitoneum, PVT, and renal failure ( *p* = 0.441, 0.946, 0.996, 0.858, and 0.460, respectively).

**Fig. 1:**
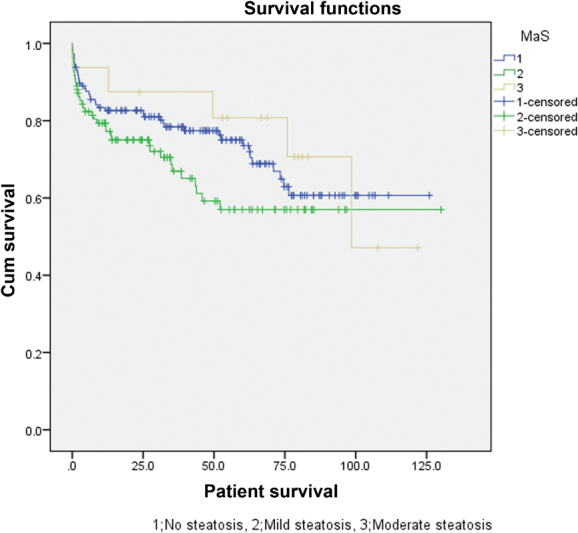
Survival plot of the patients in the macrosteatosis groups.

**Fig. 2:**
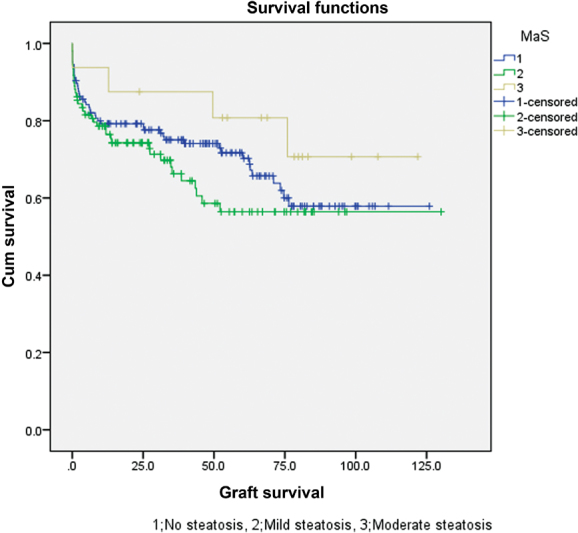
Survival plot of the grafts in the macrosteatosis groups.

As described in Table 4 , there were no significant differences among the 1-, 3-, and 5-year patient and graft survivals among the MaS groups ( *p* = 0.184 and 0.262, respectively). Survival plots of patient and graft survivals are shown in Figs. 1 and 2 , respectively.

**Table 4:** Patients and grafts survivals

Type of steatosis	Macrosteatosis ( *n* = 271)			Microsteatosis ( *n* = 271)		
Degree of steatosis	No *n* = 146	Mild *n* = 109	Moderate *n* = 16	No *n* = 175	Mild *n* = 77	Moderate *n* = 19
Patient survival	*p* = 0.184			*p* = 0.347		
1-year	83	77	94	84	73	89
3-year	78	67	88	77	68	83
5-year	75	57	81	72	61	76
Graft survival	*p* = 0.262			*p* = 0.459		
1-year	79	77	94	81	71	89
3-year	75	66	88	75	67	83
5-year	72	56	81	69	60	76

#### Impact of Graft MiS on Post LT Outcomes

As summarized in Table 3 , a significant difference was noticed in the grade V complication rate among the MiS groups ( *p* = 0.020), with a higher rate in the mild group (19.5%) compared with the control (nonsteatotic) and moderate groups (8.0 and 5.3%, respectively). This difference emerged between the mild and control groups only ( *p* = 0.009) with no differences found between the moderate and mild groups ( *p* = 0.136) or the moderate and control groups ( *p* = 0.671). No statistical differences were observed in the grade III and IV complications rate ( *p* = 0.640 and 0.186, respectively).

Regarding the overall complications encountered after LT ( Table 3 ), five cases of PNF were observed in both the mild (6.5%) and the control (2.9%) groups, with no PNF in the moderate group. These results were not significantly different ( *p* = 0.250). Additionally, there was a higher rate of EGD in the moderate group of patients (52.6%) than in the mild (35.1%) and control (34.3%) groups, respectively but these results were not significant ( *p* = 0.282). Like MaS, the ACR rate was higher in the control and mild groups than in the moderate group (39, 32.5, and 15.8%, respectively) but the differences were not significant ( *p* = 0.111).

Within the first 6 postoperative months, a higher frequency of HCV recurrence was observed in the control (9.7%) and mild (9.1%) MiS groups; no cases were observed in the moderate group ( *p* = 0.366). Moreover, nearly equal results were obtained among all MiS groups in regard to the rates of postoperative HAT, PVT, ascites, biliary leakage, biliary strictures, haemoperitoneum, and renal failure ( *p* = 0.676, 0.435, 0.524, 0.137, 0.999, 0.531, and 0.734, respectively).

**Fig. 3:**
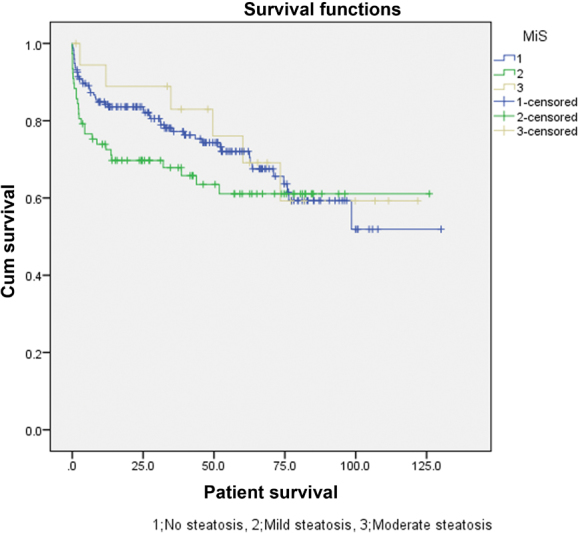
Survival plot of the patients in the microsteatosis groups.

**Fig. 4:**
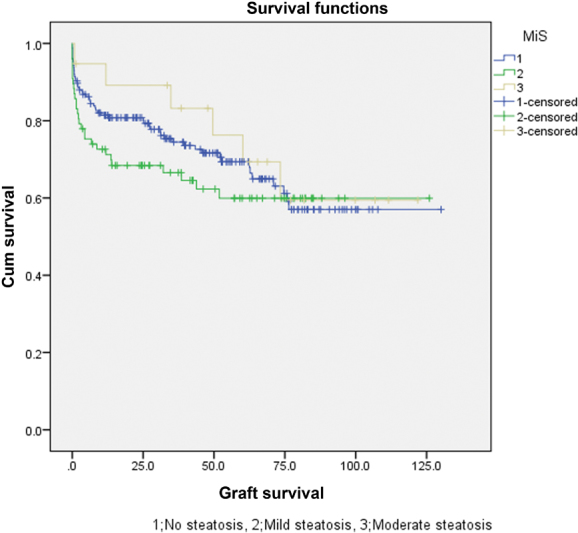
Survival plot of the grafts in the microsteatosis groups.

As described in Table 4 , there were no significant differences between the 1-, 3-, and 5-year patient and graft survivals among the MiS groups ( *p* = 0.347 and 0.459, respectively). Survival plots of patient and graft survivals are shown in Figs. 3 and 4 , respectively.

## Discussion

Several donor and recipient risk factors can contribute to post LT outcomes. Therefore in this study, the D-MELD and DRI (donor risk index) scores were carefully reviewed and found to be of lower values in cases of increased steatosis. Conversely, the recipient age and MELD score were not greatly considered in relation to the degree of steatosis. Furthermore, CIT and TIT were not considered in regard to the degree or type of steatosis, trending toward a longer duration in the moderate groups of both categories. The maximum CIT in all study groups did not exceed 10 hours.

From the results, it seems that the steatosis type or degree were not the main risk factors for post LT complication rate. Additionally, Wong et al demonstrated no significant difference in grade III, IV, and V complications between severely steatotic grafts versus a control group. 7 Moreover, Westerkamp et al reported a significant increase only in the grade IV complication rate in a moderate MaS group compared with a control group (53 vs. 13%, respectively). The majority of these complications were related to single organ failure including respiratory or renal insufficiency. 21

Although the rates of EGD paralleled the degree of steatosis in MaS and MiS and were the highest in the moderate groups (56.2 and 52.6%, respectively), no statistical significance was observed. Similarly, no significant differences were noted in the MaS and MiS groups in regard to postoperative PNF, retransplantation, PVT, HAT, haemoperitoneum, renal insufficiency, or ascites. Likewise, previous reports described early poor liver function and bilirubin levels in the moderate graft steatosis group within the first 2 days after LT. These undesirable outcomes became comparable with the control groups on POD 7 without any significant differences in the rates of EGD, PNF, retransplantation, or vascular complications. 1 21 Conversely, a recent systematic review reported a significantly increased rate of EGD in the moderate steatosis group when compared with nonsteatotic groups. In addition, a trend toward a higher rate of PNF in these groups was described but it lacked statistical significance. 22 Notably, this review demonstrated that there were large heterogeneities among the included studies in regard to the definition of PNF and EGD. Additionally, the studies that showed these significant results had few participants.

With regard to biliary complications, Frongillo et al reported an increased incidence of ischemia time biliary lesions in a mild/moderate group of steatosis. 23 In line with this report, an Italian multicentre study concluded that MaS > 25% was an independent risk factor for predicting posttransplant biliary complications. 24 Both studies attributed these results to the compression of the hepatic sinusoidal space by fatty vacuoles, causing partial, or complete obstruction with a subsequent compromise of the hepatic microcirculation which led to a reduction in tolerance to Ischaemia/Reperfusion (I/R) injury and increased susceptibility to infections. Conversely, our analysis demonstrated no significant variations among patients who received steatotic livers in either category during the first 6 postoperative months which was similar to the results of previous studies. 1 21 We supposed that differences in the rate of biliary complications were due to the variance between group numbers and this was one of the limiting factors in our study.

Interestingly, it was noticed that the ACR rates were inversely proportional to the degree of steatosis in both categories, being the lowest among the moderate groups (18.8 and 15.8%, respectively) compared with the mild (32 and 32.5%, respectively) and control groups (39.7 and 39%, respectively). However, there was no statistical difference in either category ( *p* = 0.161 and 0.111, respectively). Cho et al reported that 16.7% of patients who received nonsteatotic grafts had ACR after LT compared with 5.4% of the recipients of steatotic livers. 25 Similarly, Hejlova et al demonstrated a higher rate of ACR among patients who received grafts with no steatosis (6.5%) than among those who received steatotic grafts (4.4%). 26 In addition, Subramanian et al reported that non HCV recipients who received moderate/severe MaS grafts experienced a lower rate of ACR (16.7%) than did the mild (30%) and control (36%) groups. 27 The steatotic livers may have been preconditioned by multiple previous liver insults during the pathogenesis of steatosis. Therefore, these grafts could greatly tolerate the inflammation elicited by the disparity between the donor and recipient.

In addition, no cases in the moderate groups of MaS or MiS had HCV recurrence within the first 6 months post LT but there was not a significant difference when compared with the mild and control groups of each category ( *p* = 0.423 and 0.366, respectively). Similarly, Botha et al documented that mild and moderate graft MaS has no impact on HCV recurrence after LT for HCV related cirrhosis. Donor age and CIT were the likely risk factors associated with the increase of HCV recurrence. 28

In our study, the 1, 3, and 5-year patient and graft survival rates were equal in the moderate groups and were 94, 88, and 81%, respectively, in the MaS category and 89, 83, and 76%, respectively, in the MiS category. In comparison with the mild and control groups in each category, no statistical significance was observed.

Some limitations are considered in light of these results. First, the present analysis was derived from a retrospective study. Second, there was a low number of moderate groups of steatosis in both categories which could lead to a *β* error. Finally, in this study, we have demonstrated excellent post LT short and long-term outcomes using grafts with mild and moderate degrees of steatosis with nearly equal results with regard to the type of steatosis.

Additionally, possible advantages of the steatotic grafts have emerged in the form of decreasing the ACR rate postoperatively. Therefore, these grafts should be considered normal grafts and research should now be directed toward the use of severely steatotic livers. Moreover, the D-MELD score is a trustworthy tool for assigning donor–recipient risk factors that can be used safely in marginal graft allocation.

## Conclusion

Excellent post LT long-term outcomes using grafts with mild and moderate steatosis were determined. Further studies are needed to evaluate the newly proposed relationship between ACR and steatosis.
